# Mechanisms Involved in Cell Wall Remodeling in Etiolated Rice Shoots Grown Under Osmotic Stress

**DOI:** 10.3390/life15020196

**Published:** 2025-01-28

**Authors:** Kazuyuki Wakabayashi, Motomi Shibatsugu, Takayuki Hattori, Kouichi Soga, Takayuki Hoson

**Affiliations:** 1 Department of Biological Sciences, Graduate School of Science, Osaka Metropolitan University, Sumiyoshi-ku, Osaka 558-8585, Japansoga@omu.ac.jp (K.S.); 2 Department of Biology, Faculty of Science, Osaka City University, Sumiyoshi-ku, Osaka 558-8585, Japan; 3 Department of Biological Sciences, Graduate School of Science, Osaka City University, Sumiyoshi-ku, Osaka 558-8585, Japan

**Keywords:** BAHD acyltransferase, cell wall-bound phenolic acids, cell wall peroxidase, mechanical properties of the cell wall, *Oryza sativa*, phenylalanine ammonia-lyase, polyethylene glycol

## Abstract

Osmotic stress impacts the cell wall properties in plants. This study aimed to elucidate the mechanisms involved in cell wall remodeling in etiolated (dark-grown) rice (*Oryza sativa* L.) shoots grown under polyethylene glycol (PEG)-induced osmotic stress conditions. Shoot growth was inhibited by 70% by the treatment with 60 mM PEG for 2 days. However, when the stressed seedlings were transferred to a solution without PEG, their shoot growth rate increased significantly. A measurement of the cell wall mechanical properties revealed that the cell walls of the stressed shoots became looser and more extensible than those of unstressed shoots. Among the cell wall constituents, the amounts of cell wall-bound phenolic acids, such as ferulic acid (FA), *p*-coumaric acid (*p*-CA), and diferulic acid (DFA), per shoot and per unit of matrix polysaccharide content were significantly reduced in the stressed shoots compared to those in the unstressed shoots. Concerning the formation of cell wall-bound phenolic acids, the activity of cell wall-bound peroxidase (CW-PRX) per unit of cell wall content, which is responsible for the coupling reaction of FA to produce DFA, was 3.5 times higher in stressed shoots than in unstressed shoots, while the activity was reduced by 20% on a shoot basis in stressed shoots compared to that in unstressed shoots. The expression levels of the major class III peroxidase genes in stressed shoots were either comparable to or slightly lower than those in unstressed shoots. Conversely, the phenylalanine ammonia-lyase (PAL) activity, which contributes to the biosynthesis of FA and *p*-CA, was reduced by 55% and 30% on a shoot and unit-of-protein-content basis, respectively, in stressed shoots compared to that in unstressed shoots. The expression levels of abundantly expressed PAL genes decreased by 14–46% under osmotic stress. Moreover, the gene expression levels of specific BAHD acyltransferases, which are responsible for the addition of FA and *p*-CA to form ester-linked moieties on cell wall constituents, decreased by 15–33% under osmotic stress. These results suggest that the downregulation of the expression of specific PAL and BAHD acyltransferase genes in osmotically stressed rice shoots is responsible for a reduction in the formation of cell wall-bound phenolic acid monomers. This, in turn, may result in a decrease in the levels of DFAs. The reduction in the formation of DFA-mediated cross-linking structures within the cell wall may contribute to an increase in the mechanical extensibility of the cell wall. The remodeling of cell walls in an extensible and loosened state could assist in maintaining the growth capacity of etiolated rice shoots grown under osmotic stress and contribute to rapid growth recovery following the alleviation of osmotic stress.

## 1. Introduction

The cell wall encases the individual protoplasts and provides mechanical strength to plant cells. The cell wall exerts a direct influence on the shape and size of plant cells, and thus, it plays a crucial role in regulating the growth and morphogenesis of the plant body [1,2,3,4]. In addition to the importance of the cell wall in the regulation of plant growth and morphogenesis, it has been demonstrated that environmental stresses, particularly abiotic stresses, induce alterations in the cell wall structure and metabolism in many plant species [5,6,7,8]. For decades, cell wall remodeling in response to abiotic stresses has been regarded as a part of the process of stress acclimation and tolerance mechanisms.

The chemical properties of the cell wall, such as the quantity and structure of cell wall constituents, are considered key factors in determining its mechanical properties [9,10,11,12,13]. The cell wall of growing plant tissues consists of the primary cell wall, which is predominantly composed of various matrix polysaccharides and cellulose [1,2,9]. In addition to polysaccharide constituents, the primary cell wall of gramineous plants (Poaceae) such as rice and wheat contains a significant amount of phenolic acid monomers such as ferulic acid (FA) and *p*-coumaric acid (*p*-CA), which are ester-bound to matrix polysaccharides [14,15]. Within the cell wall, some FA residues undergo a coupling reaction to produce diferulic acid (DFA), forming cross-linkages between matrix polysaccharides [15,16,17]. The formation of DFA-mediated cross-linkages, known as the ferulate network, significantly influences the mechanical properties of the cell wall in gramineous seedlings [18,19,20].

The formation of the ferulate network results from a series of biochemical processes. Phenolic acid monomers, such as FA and *p*-CA, are synthesized via the phenylpropanoid pathway. The first step of this pathway is catalyzed by phenylalanine ammonia-lyase (PAL), which plays a rate-limiting role in the process [21,22,23]. Subsequently, the synthesized FA and *p*-CA residues are incorporated into cell wall constituents. The BAHD acyltransferases (acyl-CoA transferases) are considered to be responsible for the incorporation of FA and *p*-CA into cell wall constituents in gramineous plants [24,25,26]. Within the cell wall, cell wall-bound peroxidase (CW-PRX) catalyzes the coupling reaction of FA residues bound to matrix polysaccharides, resulting in the formation of DFA [15,27,28].

Osmotic stress caused by drought, desiccation, and salinity reduces the water uptake into plant tissues, severely affecting the plant life cycle. A significant effect of osmotic stress is the inhibition of plant organ growth [29,30]. This stress alters metabolic and biochemical processes, leading to physiological and morphological changes in plants [29,30,31]. It has been shown that osmotic stress exerts a substantial influence on the metabolic processes of cell wall constituents, including polysaccharides and phenolic components, in plants, resulting in alterations to cell wall architecture [5,6,7]. In relation to the effect of osmotic stress on cell wall-bound phenolic acids in gramineous plants, Hura et al. [32] observed that, in a winter triticale genotype exhibiting the pronounced inhibition of leaf growth (dry mass increase) under water deficit conditions, the level of cell wall-bound FA in leaves was significantly elevated in response to drought stress. Furthermore, in the case of the salt-sensitive maize genotype, the amounts of cell wall-bound FA and DFA in elongating leaf blades were found to increase in response to salt stress [33]. In contrast, two inbred lines of maize displaying divergent tolerance to drought stress exhibited no significant changes in the amounts of cell wall-bound *p*-CA, FA, or DFA in their stems when subjected to drought stress [34]. Our previous study showed that polyethylene glycol (PEG)-induced osmotic stress for several days suppressed cell wall stiffening and the formation of cell wall-bound phenolic acids, including DFA, in the shoots of etiolated wheat seedlings [35]. Our results suggest that the suppression of ferulate network formation helps maintain the mechanical extensibility of the cell wall in osmotically stressed wheat shoots.

Rice, along with wheat and maize, is one of the most important cereal plants worldwide, and it is cultivated in diverse geographical regions, including Asia. The genome size of rice is smaller than that of wheat and maize, and its complete genome sequence was determined after that of Arabidopsis. Since then, rice has emerged as a model cereal plant for extensive studies of plant biology due to the availability of its genetic information [36]. In the present study, the effects of PEG-induced osmotic stress on the mechanical and chemical properties of the cell wall were re-examined using etiolated rice seedlings. The mechanisms involved in the regulation of ferulate network formation in stressed rice shoots were elucidated by examining the gene expression levels of PAL, BAHD acyltransferases, and CW-PRX, and the activities of PAL and CW-PRX.

## 2. Materials and Methods

### 2.1. Plant Materials and Growth Conditions

Caryopses of rice (*Oryza sativa* L. cv. Koshihikari) were sterilized with a 1% (*v*/*v*) sodium hypochlorite solution and then soaked for 1 day at 25 °C in the dark. The imbibed caryopses were grown on moistened filter paper containing deionized water for 2 days at 25 °C in the dark. The 2-day-old seedlings were transferred to filter paper containing deionized water or different concentrations of a PEG 4000 (average molecular weight, 3000; FUJI FILM Wako Pure Chemical Co., Osaka, Japan) solution, and grown for 2 days at 25 °C in the dark. In the transplant experiment, the 2-day-old seedlings were grown in deionized water or a 60 mM PEG solution as described above. After 2 days, half of the PEG-treated seedlings were transferred to deionized water and then grown for a further 1 day. On the days after planting, shoots consisting of the coleoptile and first leaf were excised from the seedlings and their fresh weights were measured using an electric balance. Then, the shoot lengths were measured with a ruler. All the manipulations were performed under dim green light (ca. 0.09 μmol/m^2^/s at handling level).

### 2.2. Measurement of the Mechanical Properties of the Cell Wall

The measurements of the mechanical properties of the shoot cell wall were performed according to the method by Wakabayashi et al. [20]. The shoots excised from the rice seedlings were immediately boiled in 80% (*v*/*v*) ethanol. Before performing the measurement procedure, the ethanol-fixed shoots were rehydrated with deionized water. The mechanical properties of the cell wall were measured using a tensile tester (RTM-25; Toyo Baldwin Co., Tokyo, Japan). The middle region of the shoot was fixed between two clamps 2 mm apart, and stretched by lowering the bottom clamp at a speed of 20 mm/min until a load of 12 g (maximum stress) was produced. The cell wall extensibility (μm/g) was determined by measuring the rate of the increase in stress just before it reached the maximum stress. In addition to the cell wall extensibility, a stress–relaxation parameter, the minimum stress–relaxation time (T_0_), was calculated according to the equation reported by Yamamoto et al. [37]. With regard to the methodology employed in the stress–relaxation analysis, it has been observed that, when a specimen derived from plant organs is stretched and the strain is maintained, the stress within the specimen undergoes a reduction. The T_0_ value represents the time of the initial decay of the stress [38].

### 2.3. Determination of Cell Wall Constituents

The determination of the cell wall constituents in rice shoots was conducted as previously described [20]. The quantification of the cell wall constituents was conducted using twelve shoots for each sample. Briefly, the ester-linked phenolic acids were extracted from cell wall preparations with a 1 M NaOH solution. Then remaining residue was treated with a 17.5% NaOH solution containing 0.02% NaBH_4_. The alkali-insoluble residue was designated as cellulose. The ester-linked phenolic acids in the 1 M NaOH extract were recovered in ethyl acetate by acidification. The remaining solution was combined with the 17.5% NaOH extracts and designated as the matrix polysaccharide fraction. The total sugar contents in the matrix polysaccharide and cellulose fractions were measured by the phenol-sulfuric acid method and expressed as glucose equivalents. Ester-linked phenolic acids liberated from the cell wall were analyzed using an HPLC system equipped with a reversed-phase column and a photodiode array detector, as previously described [20].

### 2.4. Determination of CW-PRX and PAL Activities

After excision, the shoots were immediately frozen with liquid nitrogen and then kept at –80 °C. The activities of PAL and CW-PRX were assayed according to a method previously described [20]. For the CW-PRX activity assay, frozen shoots (15 control shoots or 25 PEG-treated shoots for each sample) were homogenized in ice-cold 10 mM sodium phosphate buffer, with a pH of 6.0. The cell wall residues were collected on a polypropylene mesh (32 μm pore size) and thoroughly washed with the same buffer. Peroxidases ionically bound to the cell wall were extracted using a high concentration of NaCl. The washed cell wall residues were suspended in the same buffer containing 1.5 M NaCl and kept for 24 h at 4 °C. After centrifugation at 10,000× *g*, the CW-PRX activity was measured using the supernatant and FA as a substrate. The reaction mixture (2 mL) contained 50 μL of the extract, 1 mL of 0.2 mM FA, 200 μL of 50 mM hydrogen peroxide, 250 μL of water, and 500 μL of 200 mM sodium phosphate buffer (pH of 6.0). For the quantification of the enzyme activity, the oxidation of FA was measured spectrophotometrically following the decrease in absorbance at 310 nm in a reaction mixture between 0.5 and 1.0 min after the addition of hydrogen peroxide at 25 °C.

For the PAL activity assay, the frozen shoots (9 shoots for each sample) were homogenized with ice-cold 100 mM potassium borate buffer (pH of 8.8) containing 2 mM mercaptoethanol using a mortar and pestle. After centrifugation at 10,000 g, the supernatant was used for the PAL assay. The reaction mixture (2 mL) contained 0.5 mL of 4 mM L-phenylalanine, 0.5 mL of the extract, and 1 mL of the same buffer, and was incubated at 37 °C for 60 min. The reaction was terminated by adding 0.1 mL of 5 N HCl to the mixture, and then the mixture was extracted with ethyl acetate. The amount of *t*-cinnamic acid produced by PAL was determined using the HPLC system [20]. The enzyme activity was expressed as the amount of *t*-cinnamic acid produced during the incubation. The protein content in the extract was determined using a protein assay kit (Bio-Rad Lab. Inc., Hercules, CA, USA).

### 2.5. Gene Expression Analysis

The shoots utilized in the microarray analysis were collected from three replicated cultivations. Shoots excised from seedlings (approximately 80 mg in fresh weight for each sample) were immediately frozen with liquid nitrogen and kept at –80 °C. The extraction of RNA from the rice shoots and the microarray analysis using RNA were performed by the custom service of the Bio-Medical Dept., Kurabo Industries Ltd. (Osaka, Japan) with the Rice (US) Gene 1.0 ST Array using Affymetrix GeneChip^®^. The gene expression data were normalized with the Affymetrix Expression Console software (version 1.3.0). The expression levels of the genes were analyzed using the values of fluorescence intensities obtained through the microarray analysis. For the PAL, class III peroxidase, and BAHD acyltransferase genes in rice plants, information such as the gene name and RAP-IDs was obtained from the Rice Annotation Project Database (rap-db; https://rapdb.dna.affrc.go.jp, accessed on 6 December 2024) and Passardi et al. [39].

### 2.6. Statistical Analysis

For each measurement, the mean and the standard error of the mean (SE) were calculated. Significant differences among the treatments were analyzed with Tukey’s HSD test using the R software (version 4.1.2). The statistical analysis between the control and PEG treatment was performed with Student’s *t*-test and a one-tailed *t*-test using the Microsoft Excel software. *p*-values of <0.05 were considered to be statistically significant.

## 3. Results

### 3.1. Effects of Osmotic Stress on Shoot Growth and Mechanical Properties of the Cell Wall

**Figure 1:**
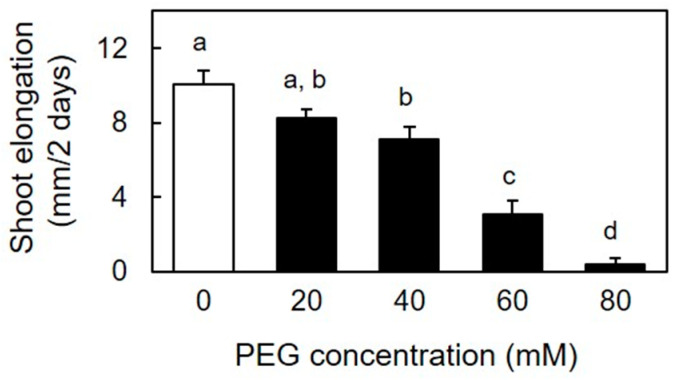
Effects of osmotic stress on elongation of etiolated rice shoots. Imbibed caryopses were initially cultivated in deionized water for 2 days, resulting in a shoot length of 2.7 ± 0.1 mm. Subsequently, the seedlings were transferred to either deionized water or various concentrations of PEG solutions and grown for an additional 2 days. The data are presented as the means ± SE (n = 15–16). Different letters above the bars denote statistically significant differences (Tukey’s HSD test, *p* < 0.05).

During the two-day incubation period, the shoot length increased by approximately 4-fold in the absence of PEG. However, the application of PEG to the roots resulted in a concentration-dependent reduction in shoot elongation (Figure 1). Treatment with 60 mM PEG for two days inhibited shoot growth by 70%, and at 80 mM PEG, shoot growth was barely observable. Given that the shoots cultivated under 60 mM PEG exhibited a diminished growth rate, but did not wilt throughout the incubation period, this concentration was selected for subsequent experiments.

The daily growth rate of stressed shoots was significantly lower than that of unstressed shoots (Table 1). However, upon transferring PEG-treated seedlings to water after two days, the shoot growth rate increased significantly and reached levels comparable to unstressed shoots (Table 1).

**Figure 2:**
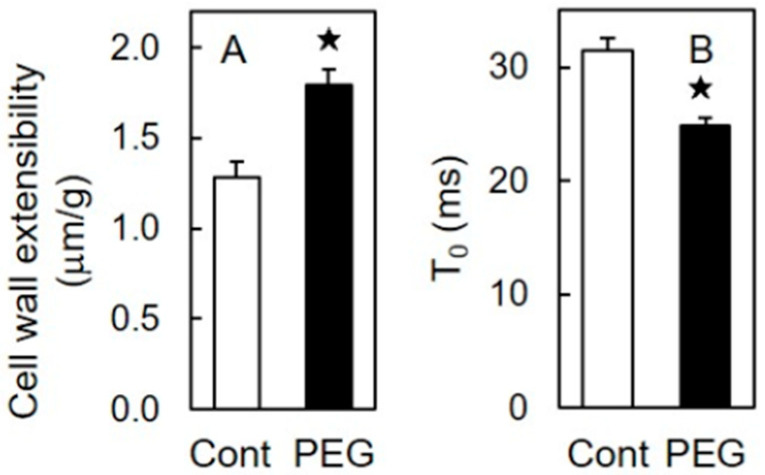
Effects of osmotic stress (60 mM PEG) on cell wall mechanical properties of etiolated rice shoots. (**A**) Cell wall extensibility; (**B**) T_0_ (minimum stress–relaxation time). The growth conditions are depicted in Figure 1. The data are presented as the means ± SE (n = 20). ★ Significant differences in the mean values between the control and PEG treatment (Student’s *t*-test, *p* < 0.05).

The mechanical properties of rice shoot cell walls were assessed using the stress–strain and stress–relaxation methods. In shoots treated with 60 mM PEG for two days, the cell wall extensibility, as measured by the stress–strain method, was significantly higher compared to that of unstressed shoots (Figure 2A). Conversely, the T_0_ value, assessed by the stress–relaxation method, exhibited the opposite trend (Figure 2B).

### 3.2. Effects of Osmotic Stress on the Amounts of Cell Wall Constituents

**Figure 3:**
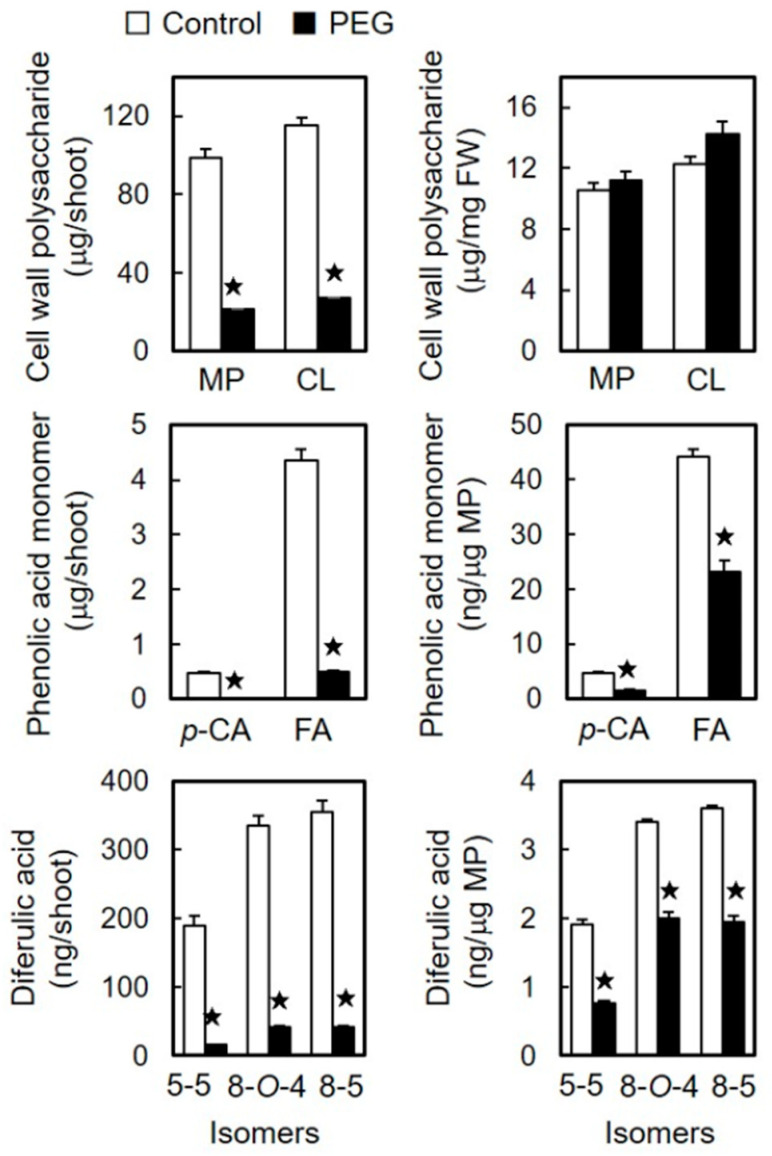
Effects of osmotic stress (60 mM PEG) on the amounts of cell wall polysaccharides, cell-wall-bound phenolic acid monomers, and cell-wall-bound diferulic acids in rice shoots. The growth conditions are depicted in Figure 1. MP, matrix polysaccharides; CL, cellulose; *p*-CA, *p*-coumaric acid; FA, ferulic acid. The data are presented as the means ± SE from three independent samples. ★ Significant differences in the mean values between the control and PEG treatment (Student’s *t*-test, *p* < 0.05).

Cell wall polysaccharides and cell wall-bound phenolic acids were quantified in shoots treated with or without 60 mM PEG for two days. The cell wall polysaccharides were fractionated into matrix polysaccharides and cellulose. The amounts of matrix polysaccharides and cellulose per shoot were significantly lower in stressed shoots compared to those in unstressed shoots (Figure 3), which was consistent with the shorter shoot length observed (Figure 1). However, when normalized per unit of shoot fresh weight, the amounts of matrix polysaccharides and cellulose in stressed shoots were nearly equivalent to those in unstressed shoots (Figure 3).

Studies have demonstrated that the cell walls of etiolated rice shoots contain three major DFA isomers: the 5–5, 8–*O*–4, and 8–5 forms [13,20,40]. Similar to the polysaccharide contents, the amounts of FA, *p*-CA, and DFA isomers per shoot were substantially lower in stressed shoots compared to those in the unstressed shoots (Figure 3). Furthermore, these phenolic acid levels were significantly reduced in stressed shoots compared to those in unstressed shoots, when normalized to the unit matrix polysaccharide content (Figure 3).

### 3.3. Effects of Osmotic Stress on Activity and Gene Expression of CW-PRX

**Figure 4:**
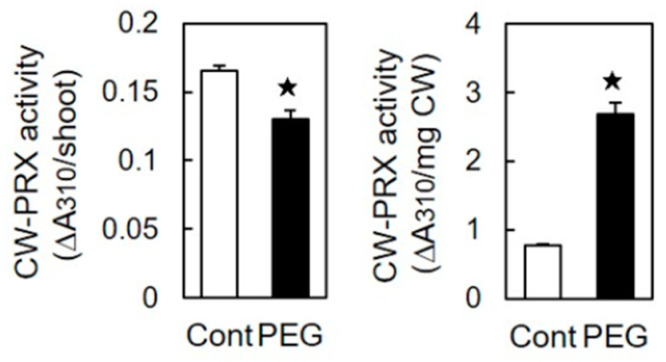
Effects of osmotic stress (60 mM PEG) on the activity of CW-PRX in rice shoots. The growth conditions are depicted in Figure 1. The CW-PRX activity was determined using FA and quantified as the reduction in absorbance at 310 nm. CW, cell wall. The cell wall content was calculated as the sum of the matrix polysaccharide and cellulose contents. The data are presented as the means ± SE from four independent samples. ★ Significant differences in the mean values between the control and PEG treatment (Student’s *t*-test, *p* < 0.05).

The CW-PRX activity per shoot decreased by 20% in stressed shoots compared to that in unstressed shoots when FA was used as a substrate in the assay. However, when normalized to the unit cell wall content, the CW-PRX activity in stressed shoots was 3.5 times higher than that in unstressed shoots (Figure 4).

Table 2 shows the expression levels of rice class III peroxidase genes. Plant peroxidases are categorized into two major groups, class I and class III. Class I peroxidases are intracellular, whereas class III peroxidases are secretory enzymes [39]. Cell wall peroxidases are classified as secretory peroxidases. To assess the CW-PRX expression levels, we analyzed the fluorescence intensity values of class III peroxidase genes obtained from a microarray analysis. According to Passardi et al. [39] and the Rice Annotation Project Database, the rice genome contains over 140 class III peroxidase genes, of which signals were detected for 119 genes, with many exhibiting very low fluorescence intensity values. Table 2 lists 35 class III peroxidase genes with intensity values higher than 50. Among these, *PRX 3*, *7*, *11*, *32*, *41*, *65*, *71*, and *Os01g0378100* showed high fluorescence intensity values. Except for *PRX 71*, the values of these genes in stressed shoots were comparable to or slightly lower than those in unstressed shoots. In contrast, the intensity values of the *PRX 110*, *111*, and *112* genes were substantially lower in stressed shoots compared to those in unstressed shoots. The total intensity values of the 35 genes (Table 2) were calculated as 8109 (100%) and 7358 (91%) for unstressed and stressed shoots, respectively.

### 3.4. Effects of Osmotic Stress on Activity and Gene Expression of PAL

**Figure 5:**
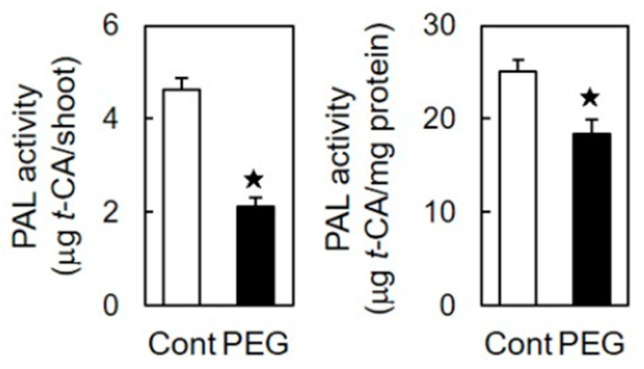
Effects of osmotic stress (60 mM PEG) on the activity of PAL in rice shoots. The growth conditions are depicted in Figure 1. The PAL activity was quantified by the production of *t*-cinnamic acid (*t*-CA) during the assay. The data are presented as the means ± SE from four independent samples. ★ Significant differences in the mean values between the control and PEG treatment (Student’s *t*-test, *p* < 0.05).

The PAL activity per shoot in stressed shoots was 55% lower compared to that in unstressed shoots (Figure 5). Moreover, the PAL activity per unit protein content was significantly reduced in stressed shoots compared to that in unstressed shoots (Figure 5).

Similar to the analysis of class III peroxidases, we examined the expression levels of PAL genes using microarray data. According to the Rice Annotation Project Database, the rice genome encompasses 11 PAL genes. Among the identified PAL genes, seven (*Os02g0626100*, *Os02g0626400*, *Os02g0627100*, *Os04g0518100*, *Os04g0518200*, *Os05g0427400*, and *Os05g0558900*) exhibited certain levels of fluorescence intensity values, while the remaining four genes displayed notably low values (Table 3). The *Os02g0626400* gene displayed a notably high fluorescence intensity value. The fluorescence intensities of these seven PAL genes were reduced by 14–46% under osmotic stress conditions (Table 3). The total intensity values of these seven PAL genes were calculated as 2636 (100%) and 1765 (67%) for unstressed and stressed shoots, respectively.

### 3.5. Effects of Osmotic Stress on Gene Expression of BADH Acyltransferases

The expression levels of BADH acyltransferase genes were examined using microarray data. The Rice Annotation Project Database indicates that the rice genome encompasses 20 BADH acyltransferase genes, of which signals were detected for 14 genes, but the *OsAT6*, *OsAT18*, and *OsAT19* genes exhibited notably low fluorescence intensity values (Table 4).

The fluorescence intensity values of the *OsAT3*, *OsAT4*, *OsAT5*, *OsAT7*, *OsAT8*, *OsAT9*, *OsAT10*, and *OsAT15* genes were reduced by 13–33% under osmotic stress conditions, while the values of the *OsAT1*, *OsAT2*, and *OsAT12* genes in stressed shoots were comparable to or higher than those in unstressed shoots (Table 4). The total intensity values of these 14 BADH acyltransferase genes were calculated as 1114 (100%) and 961 (86%) for unstressed and stressed shoots, respectively.

## 4. Discussion

The cell wall extensibility (Figure 2A), a parameter from a stress–strain analysis, reflects the capacity of the cell wall to extend, with higher values indicating a higher extension capacity [9]. The T_0_ value (Figure 2B), a parameter from a stress–relaxation analysis, indicates the degree of cell wall loosening; an increase in the T_0_ value suggests stiffening, whereas a decrease indicates loosening [38]. The cell wall extensibility of PEG-treated shoots was significantly higher than that of unstressed shoots (Figure 2A), whereas the T_0_ value of stressed shoots was significantly lower (Figure 2B). These results indicate that the cell wall of osmotically stressed rice shoots is more extensible and looser compared to that of unstressed shoots. The loosened and extensible state of the cell wall in stressed shoots likely contributes to the substantial increase in the shoot growth rate following relief from osmotic stress (Table 1). Similar results have been reported in etiolated wheat seedlings [35]. In contrast, previous studies have noted that water deficit stress often leads to cell wall stiffening in plants [5,41]. Thus, the effects of osmotic stress on the mechanical properties of the plant cell wall can vary depending on the stress intensity, duration, and cultivation conditions, such as the presence or absence of light exposure.

Changes in the cell wall constituents quantitatively influence the mechanical properties of the cell wall. The amount of cell wall polysaccharides per unit fresh weight reflects the proportion of cell walls in plant tissues. Studies have demonstrated that an increase in the proportion of cell walls results in cell wall stiffening (reduced cell wall extensibility) [13], whereas a decrease leads to decreased cell wall stiffness [11]. Based on unit fresh weight, the quantities of matrix polysaccharides and cellulose in stressed shoots were comparable to those in unstressed shoots (Figure 3), indicating that the proportion of cell walls was similar in stressed and unstressed shoots. These findings indicate that the quantity of cell wall polysaccharides may not be the sole determinant influencing the construction of the looser and more extensible cell walls observed in osmotically stressed shoots.

In gramineous seedlings, DFA-mediated cross-linkages between matrix polysaccharides play a key role in regulating the mechanical properties of the cell wall [18,19,20]. The results that the amounts of DFA isomers per unit matrix polysaccharide content were significantly lower in stressed shoots compared to those in unstressed shoots (Figure 3) indicate a reduction in the concentration of DFA-mediated cross-linkages in stressed shoot cell walls. Thus, the loosened and extensible state observed in stressed shoots is likely due to the decreased formation of the ferulate network within the cell wall. The reduction in DFA levels helps maintain the cell wall in a more extensible and loosened state in gramineous shoots [19,20,35].

The coupling process of FA catalyzed by CW-PRX plays a crucial role in regulating DFA formation [27,28,42]. A substantial increase in the CW-PRX activity during rice shoot development was closely correlated with DFA accumulation [40]. Contrary to the expectation that osmotic stress would decrease the CW-PRX activity in the cell wall, osmotic stress substantially increased the activity per unit cell wall content (Figure 4). Similarly, Lin and Kao [43] reported an increase in the CW-PRX activity per unit dry weight in the roots of rice seedlings grown under mannitol-induced osmotic stress conditions. These results suggest that osmotic stress enhances the CW-PRX activity within the cell wall architecture in gramineous seedlings. Interestingly, osmotic stress did not affect the ratio of total DFA isomers to FA content, which remained consistent at 0.20 in both stressed and unstressed shoots (calculated from Figure 3). This result suggests that, despite the increased CW-PRX activity, DFA formation was not promoted under osmotic stress. It is hypothesized that FA residues capable of forming DFA within the cell wall architecture may be limited, as DFA structures require a specific spatial configuration between FA residues [16,17,24]. Thus, a significant increase in the CW-PRX activity may not stimulate DFA formation when FA residues are reduced in the cell wall (Figure 3). In rice, class III peroxidases form a large multigenic family. Overall, the total fluorescence intensity value of the 35 detectable genes was slightly lower in stressed shoots than in unstressed shoots (Table 2). This result is in accordance with the observation that the CW-PRX activity per shoot was reduced by approximately 20% in stressed shoots compared to that in unstressed shoots (Figure 4). Thus, these results suggest that PEG-induced osmotic stress has minimal impact on the overall expression of class III peroxidase genes and the gross activity of CW-PRX in etiolated rice shoots. On the other hand, osmotic stress likely downregulates the expression of the *PRX 110*, *111*, and *112* genes (Table 2).

Phenolic acid monomers, such as FA and *p*-CA, are synthesized through the phenylpropanoid pathway, with PAL playing a rate-limiting role [21,22,23,44]. The transcriptional regulation of PAL genes is pivotal in modulating PAL activity and, therefore, it influences the production of various phenolic compounds [22,23]. Assessments based on both the shoot and unit protein content revealed lower PAL activity levels in stressed shoots compared to those in unstressed shoots (Figure 5), aligning with the reduced levels of cell wall-bound FA and *p*-CA in stressed shoots (Figure 3). Rice PAL genes form a diverse family, and among the identified genes, seven showed detectable intensity values (Table 3). The expression levels of these abundantly expressed PAL genes uniformly decreased under osmotic stress conditions (Table 3). The total intensity value of these seven PAL genes in stressed shoots decreased by more than 30% compared to that in unstressed shoots. Moreover, in stressed shoots, the mean value (0.684) of the relative expression ratio (PEG/control in Table 3) for seven PAL genes was significantly reduced in comparison to the control (*p* < 0.05 according to a one-tailed *t*-test). These findings indicate that PEG-induced osmotic stress suppresses the expression of abundantly expressed PAL genes in rice shoots, leading to decreased PAL activity, which in turn causes a reduction in the biosynthesis of FA and *p*-CA. A proteomic study conducted on rice seedlings similarly revealed that NaCl-induced salt stress reduced the PAL protein levels [45].

BAHD acyltransferases are considered to be involved in the incorporation of phenolic acid monomers, such as FA and *p*-CA, into cell wall constituents in gramineous plants [24,25,26]. The BAHD acyltransferases were named according to four biochemically characterized enzymes: BEAT (benzylalcohol *O*-acetyltransferase), AHCT (anthocyanin *O*-hydroxycinnamoyltransferase), HCBT (anthranilate *N*-hydroxycinnamoyl/benzoyltransferase), and DAT (deacetylvindoline 4-*O*-acetyltransferase) [46]. In relation to the function of BAHD acyltransferase genes in rice, Piston et al. [47] demonstrated that the downregulation of four genes (*OsAT7*, *OsAT8*, *OsAT9*, and *OsAT10*) resulted in a reduction in the level of cell wall ester-linked FA monomers in leaves. Furthermore, the overexpression of the *OsAT10* gene in rice led to an increase in the level of cell wall-bound *p*-CA in the leaf sheath and blade, and in straw [48]. For other genes, the proteins encoded by *OsAT4* and *OsAT5* genes have been shown to have enzymatic activity to transfer *p*-CA and FA to monolignols, respectively [49,50]. These findings suggest that enzymes encoded by the *OsAT4*, *5*, *7*, *8*, *9*, and *10* genes may have a biochemical function in vivo in rice plants. The present results show that the expression levels of those *OsAT* genes were found to decrease by 15–33% under osmotic stress (Table 4). Furthermore, in stressed shoots, the mean value (0.758) of the relative expression ratio (PEG/control in Table 4) for the *OsAT4*, *5*, *7*, *8*, *9*, and *10* genes was significantly reduced in comparison to the control (*p* < 0.05 according to a one-tailed *t*-test). These results imply that the capacity of the incorporation of phenolic acid monomers into cell wall constituents may be reduced in osmotically stressed shoots. The downregulation of the expression of specific PAL and BAHD acyltransferase genes may be cooperatively involved in the suppression of the formation of cell wall-bound phenolic acid monomers, which in turn results in reduced levels of cell wall-bound DFAs in stressed rice shoots.

The precise mechanism through which PEG-induced osmotic stress downregulates the expression of specific PAL and BAHD acyltransferase genes remains unclear. It has been demonstrated that osmotic stress stimulates the production of reactive oxygen species (ROS) [30,31,51]. The overproduction and accumulation of ROS in plant tissues are considered to be stress signals, and it is probable that ROS signaling is involved in the initiation of stress-induced molecular, biochemical, and physiological responses [30,31]. In addition to ROS, abscisic acid (ABA), a plant hormone, is produced in response to osmotic stress [29,52]. Our previous study showed that the exogenous application of ABA to etiolated wheat seedlings suppressed the increment of PAL activity and the accumulation of cell wall-bound phenolic acids in shoots [53]. These findings suggest that the ROS and ABA signaling pathways may play a role in the downregulation of PAL and BAHD acyltransferase genes during osmotic stress in rice shoots.

Etiolated seedlings have not developed the capacity for photosynthesis, which results in a restricted energy supply. It can, therefore, be posited that increasing the cell wall extensibility by inhibiting the synthesis of cell wall components may be an effective strategy for maintaining the growth capacity under osmotic stress conditions while reducing the biological costs.

## 5. Conclusions

The findings of this study reveal that PEG-induced osmotic stress lowers the expression levels of specific PAL and BAHD acyltransferase genes in etiolated rice shoots, leading to a reduction in the formation of ester-linked phenolic acid monomers in their cell walls. Consequently, this decrease likely results in reduced levels of DFAs. The reduction in the formation of DFA-mediated cross-linking structures within the cell wall may enhance the capacity of the cell wall to extend, potentially aiding in maintaining shoot growth capacity during osmotic stress. In etiolated seedlings of gramineous plants, such cell wall remodeling may play a pivotal role in tolerance mechanisms against osmotic stress while reducing the biological (energetic) costs.

## Figures and Tables

**Table 1:** Effect of osmotic stress (60 mM PEG) and its relief on the growth rate of rice shoots. Rice seedlings initially grown in deionized water for 2 days were transferred to either deionized water (control) or a 60 mM PEG solution (PEG) and then grown for 3 days. In the growth recovery experiment, seedlings treated with 60 mM PEG for 2 days were subsequently transferred back to deionized water on the fourth day and grown for an additional 1 day (PEG → water). The data are presented as the means ± SE (n = 15–18). Different letters denote significant differences between values (Tukey’s HSD test, *p* < 0.05).

Treatment	Shoot Growth Rate (mm/day)		
	2 d~3 d	3 d~4 d	4 d~5 d
Control	5.13 ± 0.28 ^a^	5.05 ± 0.40 ^a^	4.62 ± 0.42 ^a^
PEG	0.63 ± 0.23 ^b^	2.13 ± 0.34 ^b^	2.11 ± 0.39 ^b^
PEG → Water			4.72 ± 0.49 ^a^

**Table 2:** Effect of osmotic stress (60 mM PEG) on the expression levels of class III peroxidase (PRX) genes in rice shoots. The growth conditions are depicted in Figure 1. The expression levels were assessed using microarray analysis data and quantified as the fluorescence intensity. Genes with a fluorescence intensity of >50 are presented. Figures in parentheses following the RAP ID indicate the OsPRX number.

Class III PRX Genes RAP ID	Signal Intensity		Ratio (PEG/Control)
	Control	PEG	
*Os01g0205900* (2)	193	135	0.70
*Os01g0263300* (3)	315	374	1.19
*Os01g0270300* (4)	160	166	1.04
*Os07g0157000* (7)	355	368	1.04
*Os01g0294700* (11)	873	796	0.91
*Os01g0326000* (12)	56	45	0.80
*Os01g0962700* (20)	95	101	1.06
*Os02g0236800* (26)	207	209	1.01
*Os02g0236600* (27)	116	109	0.94
*Os02g0833900* (32)	364	291	0.80
*Os03g0121200* (33)	216	177	0.82
*Os03g0234900* (39)	69	63	0.91
*Os03g0339300* (41)	301	335	1.11
*Os04g0465100* (55)	88	86	0.98
*Os04g0656800* (58)	129	85	0.66
*Os04g0688100* (59)	106	133	1.25
*Os05g0134400* (65)	1829	1692	0.93
*Os05g0135200* (69)	43	76	1.77
*Os05g0135500* (71)	556	375	0.67
*Os05g0499300* (74)	162	168	1.04
*Os06g0547400* (86)	171	122	0.71
*Os06g0681600* (89)	63	57	0.90
*Os06g0695500* (90)	60	42	0.70
*Os07g0104100* (97)	198	143	0.72
*Os07g0677100* (110)	68	9	0.13
*Os07g0677200* (111)	103	10	0.10
*Os07g0677300* (112)	148	7	0.05
*Os07g0694300* (116)	40	96	2.40
*Os10g0109300* (125)	107	112	1.05
*Os10g0536700* (128)	114	107	0.94
*Os11g0661600* (134)	60	71	1.18
*Os12g0191500* (137)	185	75	0.41
*Os12g0530100* (138)	59	77	1.31
*Os01g0378100*	303	335	1.11
*Os03g0434800*	197	311	1.58

**Table 3:** Effect of osmotic stress (60 mM PEG) on the expression levels of PAL genes in rice shoots. The growth conditions are depicted in Figure 1. The expression levels were assessed using microarray analysis data and quantified as the fluorescence intensity.

PAL Genes RAP ID	Signal Intensity		Ratio (PEG/Control)
	Control	PEG	
*Os02g0626100*	487	368	0.76
*Os02g0626400*	1153	702	0.61
*Os02g0626600*	10	12	1.20
*Os02g0627100*	168	91	0.54
*Os04g0518100*	294	183	0.62
*Os04g0518400*	12	15	1.25
*Os04g0518200*	210	181	0.86
*Os05g0427400*	60	38	0.63
*Os05g0558900*	264	202	0.77
*Os11g0708900*	5	6	1.20
*Os12g0520200*	19	16	0.84

**Table 4:** Effect of osmotic stress (60 mM PEG) on the expression levels of BAHD acyltransferase genes in rice shoots. The growth conditions are depicted in Figure 1. The expression levels were assessed using microarray analysis data and quantified as the fluorescence intensity. Figures in parentheses following the RAP ID indicate the OsAT number.

BAHD Acyltransferase Genes RAP ID	Signal Intensity		Ratio (PEG/Control)
	Control	PEG	
*Os01g0615300* (*OsAT1*)	203	202	1.00
*Os01g0615200* (*OsAT2*)	52	52	1.00
*Os05g0136900* (*OsAT3*)	15	13	0.87
*Os01g0291500* (*OsAT4*)	162	119	0.73
*Os05g0278500* (*OsAT5*)	46	32	0.70
*Os01g0179000* (*OsAT6*)	8	7	0.88
*Os05g0179300* (*OsAT7*)	128	106	0.83
*Os06g0595800* (*OsAT8*)	213	180	0.85
*Os01g0185300* (*OsAT9*)	31	24	0.77
*Os06g0594600* (*OsAT10*)	18	12	0.67
*Os04g0175500* (*OsAT12*)	53	67	1.26
*Os10g0108700* (*OsAT15*)	176	139	0.79
*Os10g0122500* (*OsAT18*)	5	4	0.80
*Os04g0172400* (*OsAT19*)	4	4	1.00

## Data Availability

The data presented in this study are available from the corresponding author upon request.
